# Successful clinical approach to the metastatic uterine leiomyosarcoma to the epicardium—a case report

**DOI:** 10.1186/s12872-023-03689-8

**Published:** 2024-01-13

**Authors:** Kristina Krzelj, Ante Lekic, Vlatka Reskovic Luksic, Davor Milicic, Ivana Ilic, Luka Simetic, Zrinka Starcevic Dzepina, Hrvoje Gasparovic, Bojan Biocina, Ivica Safradin

**Affiliations:** 1 Department of Cardiac Surgery, University Hospital Centre Zagreb, Kispaticeva 12, Zagreb, 10000 Croatia; 2 Department of Cardiovascular Diseases, University Hospital Centre Zagreb, Kispaticeva 12, Zagreb, 10000 Croatia; 3 Department of Pathology and Cytology, University Hospital Centre Zagreb, Kispaticeva 12, Zagreb, 10000 Croatia; 4 Department of Oncology, University Hospital Centre Zagreb, Kispaticeva 12, Zagreb, 10000 Croatia

**Keywords:** Women’s oncology, Sarcoma, Cardiac masses, Diagnosis, Treatment, Outcomes

## Abstract

**Background:**

Uterine leiomyosarcoma is a rare and aggressive tumour with a poor prognosis. Its metastases to the heart are even rarer, especially to the epicardium. The majority of reported cardiac metastases of uterine leiomyosarcoma were in the cardiac chambers or intramyocardial. Surgical resection of the uterine leiomyosarcoma in the early stages is the only definitive treatment for this disease. However, in the cases of cardiac metastasis, surgery is recommended only in emergencies and patients with expected beneficial outcomes.

**Case presentation:**

Our patient was a 49-year-old female referred to the Department of Cardiac Surgery for scheduled surgery of pericardial neoplasia. The patient underwent a hysterectomy and adnexectomy three years prior owing to the uterine leiomyosarcoma. A regular follow-up magnetic resonance imaging of the abdomen and pelvis discovered neoplasia in the diaphragmic portion of the pericardium. No other signs of primary disease relapse or metastases were found. The patient was asymptomatic. The multidisciplinary team concluded that the patient is a candidate for surgery. Surgery included diastolic cardiac arrest achievement and resection of the tumour. Macroscopically, a parietal layer of the pericardium was completely free from the tumour that invaded only the apical myocardium of the left ventricle. Completed histopathology confirmed the diagnosis of leiomyosarcoma of the uterine origin. Three months after surgery, the patient received adjuvant chemotherapy with doxorubicin and dacarbazine. One year after surgery, there are no signs of new metastases.

**Conclusions:**

Strict surveillance of patients with uterine leiomyosarcoma after successful treatment of the early stage of the disease is of utmost importance to reveal metastatic disease to the heart in a timely manner and to treat it with beneficial outcomes. Surgery with adjuvant chemotherapy might be a good approach in patients with a beneficial prognosis. From a surgical point of view, it is challenging to assess the appropriate width of the resection edges to be radical enough and, at the same time, sufficiently conservative to ensure the satisfactory postoperative function of the remaining myocardium and avoid repetitive tumour growth. Therefore, intraoperative histopathology should always be performed.

## Background

Uterine leiomyosarcoma (ULMS) is a rare and very aggressive mesenchymal tumour with poor 5-year survival rates, accounting for 1.3% of all uterine malignancies [1]. ULMS metastasizes most frequently to the lung, peritoneum, bone, and liver [2], whereas metastasis to the heart is very uncommon. ULMS diagnosis can definitely be established only after histopathological analysis because symptoms and signs of ULMS resemble benign uterine myomas. However, in patients with early surgical intervention including hysterectomy and adnexectomy, the prognosis is beneficial. In cases of ULMS metastasis to the heart, there are certain treatment challenges that should be considered in a multidisciplinary team. Hereby, we report our experience in the case of successful treatment of solitary ULMS metastasis to the visceral layer of the pericardium.

### Case presentation

**Fig. 1:**
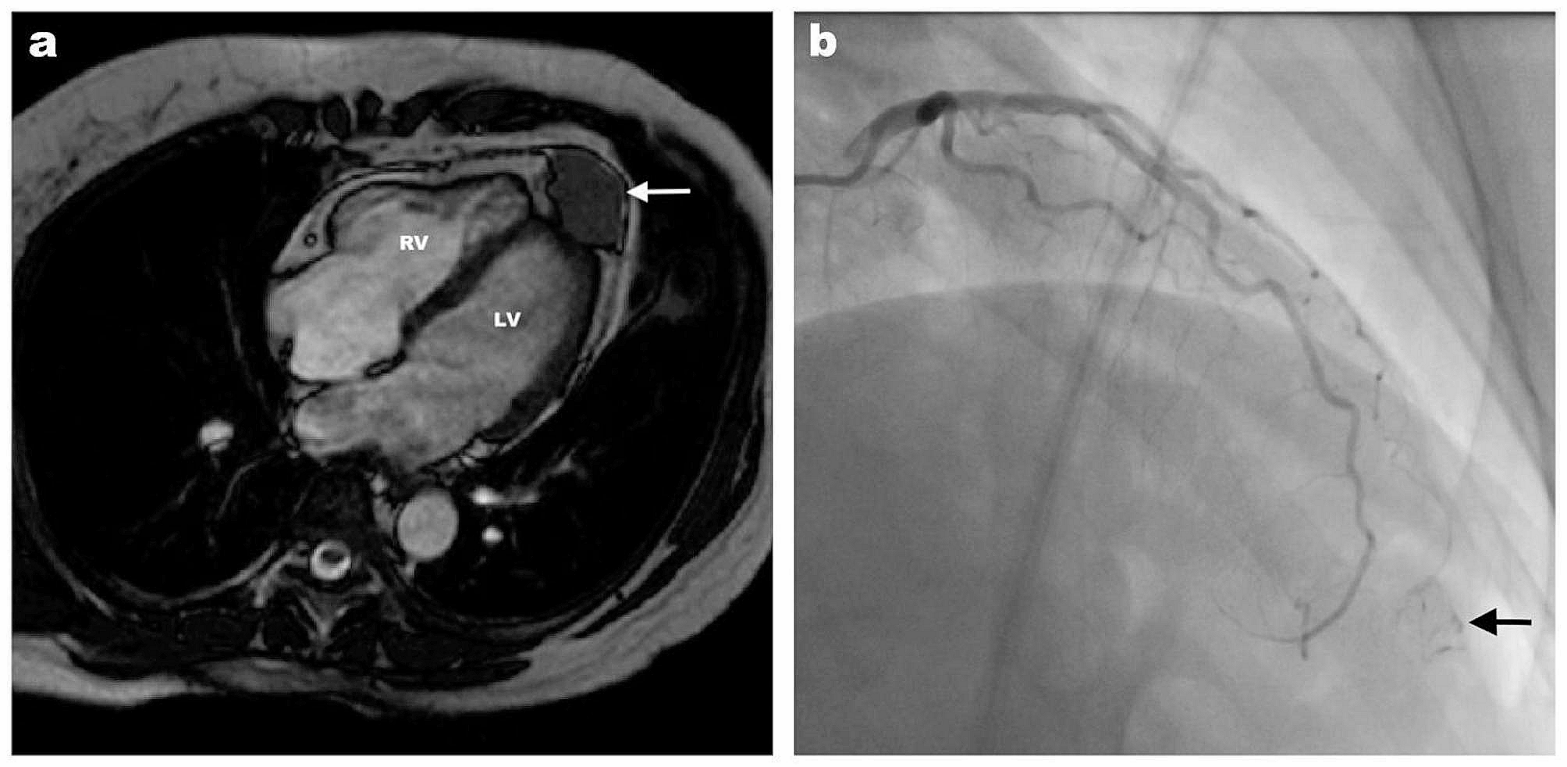
MRI of the suspected cardiac metastasis of the ULMS. **a**) A cardiac MRI shows an intrapericardial tumour (white arrow) adjacent to the apex of the left ventricle. LV—left ventricle; RV—right ventricle. **b**) Coronary artery angiography shows tumour vascular supply from the left anterior descending artery (black arrow)

**Fig. 2:**
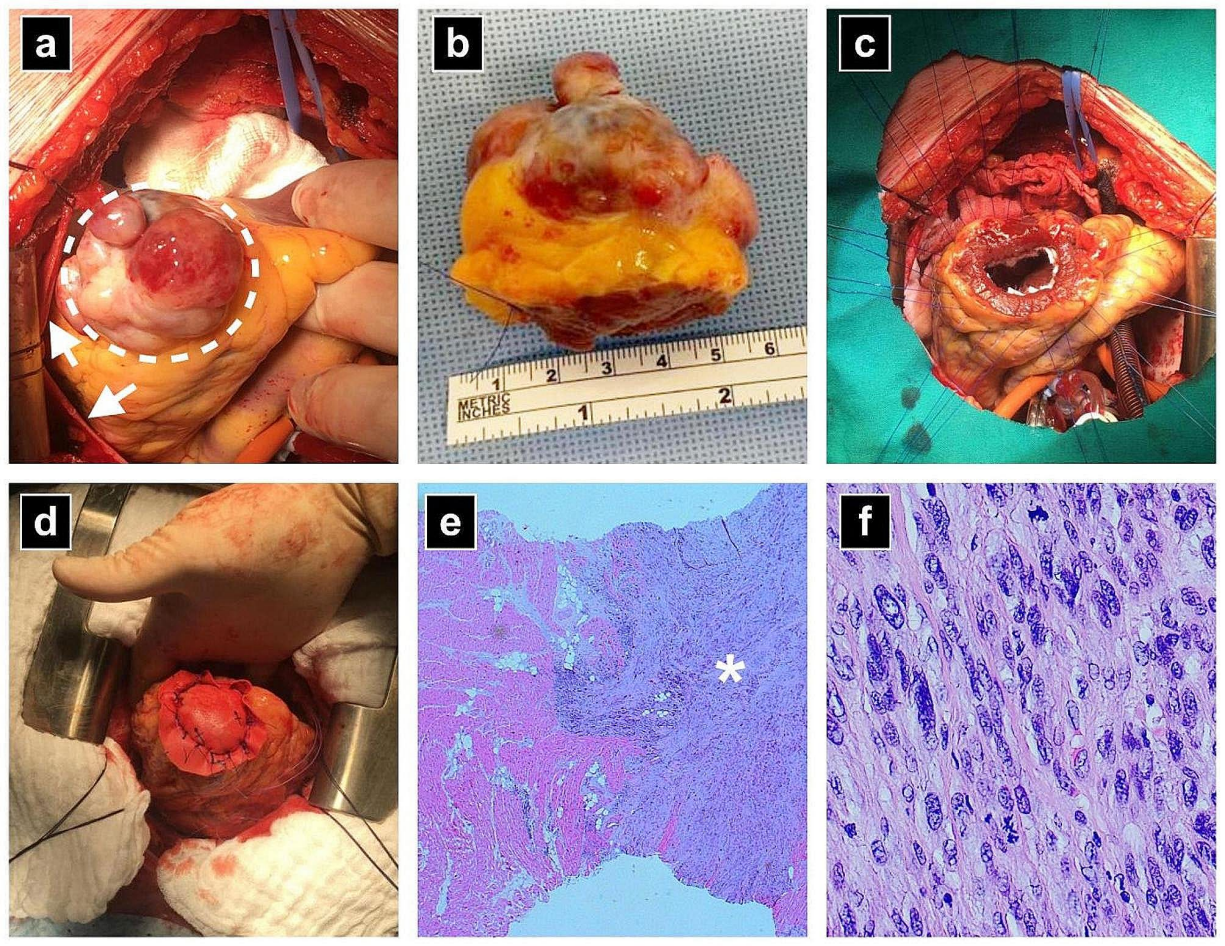
Macroscopic features of the suspected ULMS metastasis to the epicardium, surgical technique and histopathology of the tumour. **a**) Intraoperative finding - the tumour (interrupted line) is on the apex of the heart and completely free from the parietal layer of the pericardium (arrows); **b**) tumour mass after resection; **c**) defect of the left ventricle after resection of the tumour with polypropylene sutures placed around the defect; **d**) defect of the ventricle reconstructed with a polyester patch; **e**) histopathology - tumour cells (asterisk) infiltrate myocardium but not endocardium (HE staining, x2); **f**) histopathology - tumour cells are spindle-shape, pleomorphic with high mitotic activity (HE staining, x40)

Our patient was a 49-year-old female referred to the Department of Cardiac Surgery for scheduled surgery of pericardial neoplasia. Three years prior, the patient underwent a hysterectomy and adnexectomy owing to the ULMS. Considering the fact that the tumour was limited to the uterus, further chemotherapy or radiotherapy was not needed. Three months before admission to our institution, regular follow-up included magnetic resonance imaging (MRI) of the abdomen and pelvis and a computed tomography (CT) scan of the chest. The MRI of the abdomen and pelvis discovered neoplasia in the diaphragmic portion of the pericardium, whereas a CT chest scan showed the tumour mass adjacent to the apex of the heart. No other signs of primary disease relapse or metastases were found. The patient was asymptomatic, in good general condition, and the physical exam was unremarkable. After admission to our institution further workup included a heart MRI that confirmed the finding of the tumour between two layers of the pericardium (Fig. 1a) and adjacent to the apex of the heart, whereas on the performed PET/CT scan, besides the intrapericardial tumour, the presence of other metastases was excluded. Coronary angiography revealed tumour vascular supply from the left anterior descending coronary artery (LAD) (Fig. 1b). The multidisciplinary team concluded that the patient was a candidate for surgery. Surgery included diastolic cardiac arrest achievement and resection of the tumour with macroscopically healthy edges width of 5–8 mm. Macroscopically, a parietal layer of the pericardium was completely free from the tumour that invaded only the apical myocardium of the left ventricle (Fig. 2a and b). Intraoperative histopathology showed resected edges free of tumour cells. The defect of the left ventricle was reconstructed with a polyester patch and polypropylene sutures placed around the defect (Fig. 2c and d).

Intra- and the postoperative course went uneventfully. Completed histopathology confirmed the diagnosis of leiomyosarcoma (Fig. 2e and f) with positive immunohistochemical stains for oestrogen and progesterone receptors confirming the uterine origin of the tumour.

The patient was discharged from the hospital after 13 days. The control CT scans of the chest, abdomen and pelvis performed two, six and twelve months later did not show any relapse of the primary disease. Three months after the cardiac surgery, the patient received adjuvant chemotherapy with doxorubicin and dacarbazine. Consecutive control echocardiographs showed a left ventricular ejection fraction of 55% and no pericardial effusion. One year after surgery, there are no signs of new metastases, the Eastern Cooperative Oncology Group (ECOG) performance status is grade 0, whereas New York Heart Association (NYHA) functional class is I.

## Discussion

ULMS metastases to the heart are very rare and require thorough deliberation of medical treatment. Even though ULMS metastases are related to the advanced stages of the disease, [2] our case showed that after adequate treatment in the early stages, late ULMS metastases are possible, even to very uncommon sites, such as pericardium. Malignant tumours may reach the heart via hematogenous or lymphatic spread, transvenous extension or direct invasion. The malignancies that spread through the lymphatics often seed the pericardium or epicardium, whereas myocardial and endocardial metastases generally rise from the hematogenous spread [3]. The majority of reported ULMS metastases to the heart were intracavitary [4–9]. In our case, the major portion of the metastasis was epicardial, whereas only a smaller part invaded the myocardium without relation to the cardiac chambers. Although ULMS metastases to the heart are rare, we should always pay attention to this possibility in patients with ULMS. In the context of the past medical history positive for ULMS, and the fact that primary leiomyosarcomas of the heart are extremely rare and constitute less than 0.25% of all cardiac tumours, [10] it was more likely that our patient had metastatic disease rather than primary leiomyosarcoma of the heart. However, considering the unpredictable nature of malignant diseases, these two entities should be distinguished. We performed immunohistochemical staining for oestrogen and progesterone receptors that confirmed the uterine origin of the tumour.

In our case, regular follow-up ensured early detection of ULMS cardiac metastasis and early treatment before any symptoms developed. Our multidisciplinary team opted for surgery and adjuvant chemotherapy because the patient had solitary metastasis, was in good general condition without any symptoms, and was very motivated for the treatment. Surgery is generally recommended only in selected conditions - in patients with intracavitary metastases resulting in significant hemodynamic complications and in patients with solitary cardiac disease when the primary tumour is controlled, and a beneficial prognosis is expected [3]. From a surgical point of view, it is very challenging to assess the width of the resection edges to be radical enough because leiomyosarcoma is a very aggressive tumour and the remaining tumour cells along resection edges could result in tumour growth and the need for repetitive surgery. Moreover, the surgery should be sufficiently conservative at the same time to ensure the satisfactory function of the remaining ventricle postoperatively. Therefore, intraoperative histopathology might be very helpful and should always be performed to avoid excessive resection and recurrent tumour growth after surgery as well. Although our patient had late and solitary metastasis of ULMS which was completely resected, according to the current recommendations [11] the stage of the disease required adjuvant chemotherapy. This approach resulted in the absence of any new metastases in the one-year follow-up. However, current evidence for adjuvant chemotherapy in oligometastatic ULMS is weak [12, 13]. Therefore, we do not know whether chemotherapy contributed to the 1-year relapse-free survival after surgery in our patient and further studies are certainly needed to clarify this issue.

## Conclusion

Notwithstanding the ULMS by itself and ULMS metastases to the heart, especially to the visceral layer of the pericardium, are very rare, there is a certain possibility for such an appearance. Our case demonstrated that strict surveillance of patients with ULMS even after successful treatment of the early stage of the disease is of utmost importance to reveal metastatic disease to the heart in a timely manner and to treat it with beneficial outcomes. Such cases should always be carefully discussed in a multidisciplinary team in a tertiary centre, and surgery with adjuvant chemotherapy might be a good approach in patients with beneficial prognosis. Considering the surgical challenges of epicardial metastasis of ULMS, the main aim should be to ensure radical resection to avoid repetitive tumour growth and satisfactory function of the remaining myocardium. Therefore, intraoperative histopathology might contribute to surgical decision-making and is strongly recommended.

## Data Availability

Not applicable.

## References

[1] Tirumani SH, Ojili V, Shanbhogue AKP, Fasih N, Ryan JG, Reinhold C. Current concepts in the imaging of uterine sarcoma. Abdom Imaging. 2013;38. 10.1007/s00261-012-9919-x. 10.1007/s00261-012-9919-x 22699695

[2] Tirumani SH, Deaver P, Shinagare AB, Tirumani H, Hornick JL, George S, et al. Metastatic pattern of uterine leiomyosarcoma: retrospective analysis of the predictors and outcome in 113 patients. J Gynecol Oncol. 2014;25. 10.3802/jgo.2014.25.4.306. 10.3802/jgo.2014.25.4.306 PMC4195301 25142630

[3] Goldberg AD, Blankstein R, Padera RF. Tumors metastatic to the heart. Circulation. 2013;128. 10.1161/CIRCULATIONAHA.112.000790. 10.1161/CIRCULATIONAHA.112.000790 24126323

[4] Maebayashi A, Nagaishi M, Nakajima T, Hata M, Xiaoyan T, Kawana K. Successful surgical treatment of cardiac Metastasis from uterine leiomyosarcoma: a case report and literature review. J Obstet Gynecol Res. 2020;46. 10.1111/jog.14231. 10.1111/jog.14231 32166826

[5] Suraci N, Hoyos J, Baruqui D, Santana O. Right ventricular outflow tract obstruction due to a leiomyosarcoma. Ann Card Anaesth. 2020;23. 10.4103/aca.ACA_89_19. 10.4103/aca.ACA_89_19 PMC7559950 32687094

[6] Antón FM, Herraez AC, Vázquez JP, Díaz RG, Aragoncillo P, García EDR. Cardiac Metastasis from uterine leiomyosarcoma. Clin Transl Oncol. 2006;8. 10.1007/s12094-006-0186-6. 10.1007/s12094-006-0186-6 16760015

[7] Calleja AM, Wellnitz CV, Alharthi MS, Khandheria BK, Chaliki HP. Extensive cardiac metastases secondary to uterine leiomyosarcoma. J Am Soc Echocardiogr 2009;22. 10.1016/j.echo.2009.06.009. 10.1016/j.echo.2009.06.009 19647410

[8] Martin JL, Boak JG (1983). Cardiac Metastasis from uterine leiomyosarcoma. J Am Coll Cardiol.

[9] Peng YJ, Hueng GG, Lee HS. Acute Heart Failure as manifestation of metastatic uterine leiomyosarcoma to the heart and lung. Heart and Lung: Journal of Acute and Critical Care. 2004;33. 10.1016/j.hrtlng.2003.10.006. 10.1016/j.hrtlng.2003.10.006 14983139

[10] Antunes MJ, Vanderdonck KM, Andrade CM, Rebelo LS. Primary cardiac leiomyosarcomas. Ann Thorac Surg. 1991;51. 10.1016/0003-4975(91)91031-P. 10.1016/0003-4975(91)91031-p 2039335

[11] von Mehren M, Kane JM, Agulnik M, Bui MM, Carr-Ascher J, Choy E, et al. Soft tissue sarcoma, Version 2.2022. JNCCN J Natl Compr Cancer Netw. 2022;20. 10.6004/jnccn.2022.0035. 10.6004/jnccn.2022.0035 PMC10186762 35830886

[12] Pérez-Fidalgo JA, Ortega E, Ponce J, Redondo A, Sevilla I, Valverde C, et al. Uterine sarcomas: clinical practice guidelines for diagnosis, treatment, and follow-up, by Spanish group for research on sarcomas (GEIS). Ther Adv Med Oncol. 2023;15. 10.1177/17588359231157645. 10.1177/17588359231157645 PMC10052607 37007636

[13] Denschlag D, Ackermann S, Battista MJ, Cremer W, Egerer G, Fehr M, et al. Sarcoma of the Uterus. Guideline of the DGGG, OEGGG and SGGG (S2k-Level, AWMF Registry No. 015/074, April 2021). Geburtshilfe Frauenheilkd. 2022;82. 10.1055/a-1897-5124. 10.1055/a-1897-5124 PMC9715351 36467974

